# Repeat procedures after pulsed field ablation for atrial fibrillation: tertiary centre experience and meta-analysis of observational studies

**DOI:** 10.1007/s10840-026-02258-1

**Published:** 2026-02-19

**Authors:** Francis J. Ha, Sarah Paitry, Duron Prinsloo, Hui-Chen Han, Adam J Brown, Nitesh Nerlekar, Emily Kotschet

**Affiliations:** Victorian Heart Institute and Victorian Heart Hospital, Monash University, Monash Health, 631 Blackburn Road, Clayton, VIC 3168 Australia

**Keywords:** Atrial fibrillation, Pulsed field ablation, Repeat procedure, Redo ablation

## Abstract

**Background:**

Limited real-world data exist regarding pulmonary vein (PV) and posterior wall (PW) durability after index pulsed field ablation (PFA) for atrial fibrillation (AF) in patients undergoing repeat procedure.

**Methods:**

We performed a single-centre retrospective observational study of consecutive patients who underwent repeat procedure for arrhythmia recurrence after index PFA for AF and a meta-analysis of published observational studies. All patients underwent index PV ± PW isolation with Farapulse/Farawave (Boston Scientific, US). Endpoints were incidence of repeat procedure and durability of PVI and PW ablation at repeat procedure. A meta-analysis was also performed evalutaing these endpoints following PFA with the same pentaspline catheter.

**Results:**

Out of 751 index PFA procedure, 50 patients underwent repeat procedure (6.7%) at median 6.8 months (IQR 3.2–10.1). Arrhythmia recurrence was 48% AF, 36% AT/AFL and 16% both. Persistent AF at index ablation was associated with repeat procedure (*p* = 0.03). In 48 patients, PVs were durably isolated in 38%. Most common reconnection was RSPV (44%; 21/48), LSPV (40%; 19/48), RIPV (25%; 12/48) then LIPV (21%; 10/48). Superior PVs were more commonly reconnected than inferior PVs (*p* = 0.005). PW reconnection occurred in 70% (16/23). Meta-analysis of 6 further studies (*n* = 708) showed repeat procedure rate of 8.8% (95%CI 0.05–0.11). PVs were reconnected in 57% (95%CI 0.48–0.65) and PW reconnected in 55% (95%CI 0.39–0.71).

**Conclusions:**

These real world data and meta-analysis show limited PV and PW durability in patients with arrhythmic recurrence undergoing repeat procedure after PFA for AF. Additional procedural strategies are needed to enhance lesion durability from PFA.

**Graphical Abstract:**

**Figure:**
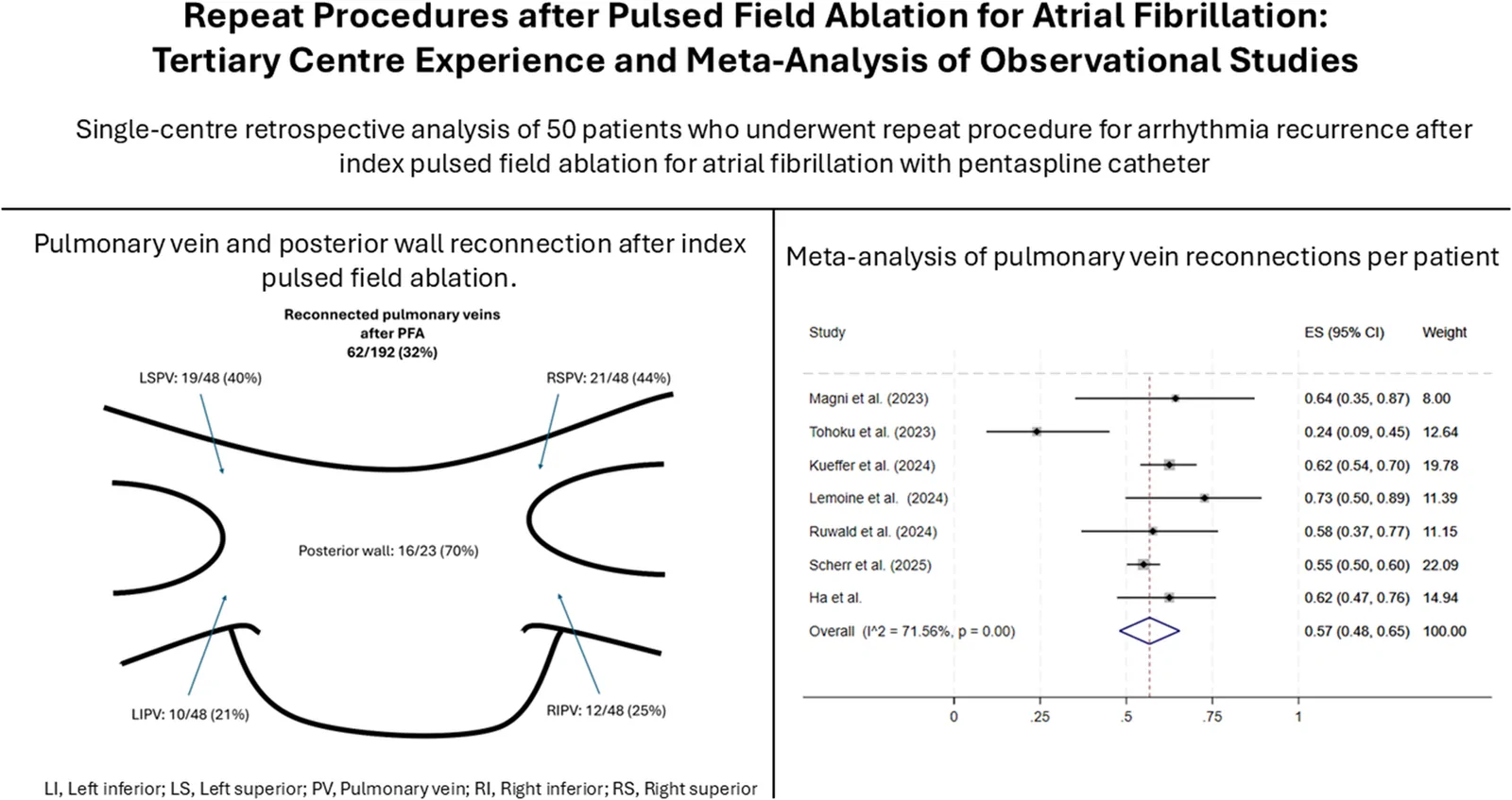

**Supplementary Information:**

The online version contains supplementary material available at https://doi.org/10.1007/s10840-026-02258-1.

## Introduction

Pulsed field ablation (PFA) has emerged as a key ablation technology for atrial fibrillation (AF) [1]. Randomized controlled trials have shown significantly reduced procedural times compared with thermal ablation while maintaining similar clinical efficacy [2, 3]. While its novelty and significant industry drive has led to widespread uptake, there is limited understanding of lesion durability at longer term follow-up. Early non-randomized trials (IMPULSE and PEFCAT) showed lesion durability of 100% in the pulmonary veins (PVs) at protocolised 3-month remapping studies after index PFA with waveform refinement [4]. Similarly, a German study reported a repeat procedure rate of 6.9% and in 25 patients, the PV durability rate was 91% at 6.1 months after index procedure [5]. Despite being only short term follow-up, such durability is still quite remarkable when compared with 90–94% PV reconnection observed in patients from the CIRCA-DOSE study using contact-force sensing radiofrequency or cryoballoon ablation catheters [6]. However, more recent registry data concerning repeat procedures after index PFA have been less optimistic. Durable vein isolation from the EU-PORIA registry was only 38% at a median of 7 months post-index procedure,^7^ while the MANIFEST-REDO registry showed durable vein isolation in 45% of patients at median 9.2 months post-index ablation [8]. Given the ongoing disparate findings, further real world clinical data regarding PV durability as well as posterior wall (PW) durability after PFA are needed.

In this study, we sought to evaluate incidence of and findings from repeat procedures after index PFA for AF at our tertiary centre. We additionally performed a meta-analysis of published observational studies to date using a specific pentaspline catheter (Farapulse/Farawave, Boston Scientific, US) to provide pooled estimates on repeat procedures after PFA.

## Methods

We performed a retrospective observational cohort study of patients who underwent repeat procedure for atrial tachyarrhythmia recurrence after previously undergoing index PFA with a pentaspline catheter for paroxysmal or persistent AF at a large tertiary centre in Australia from 2022 to 2025. Approval for this study was granted by the Monash Health Human Research Ethics Committee (RES-25-0000-748Q).

### Baseline demographics

Clinical data were extracted from electronic medical records. Baseline demographic variables included age, sex, body mass index (BMI), type of AF (paroxysmal or persistent) and medical comorbidities. The CHA₂DS₂-VASc score was calculated for each patient. Left ventricular ejection fraction (LVEF) and indexed left atrial volume (LAVI) were collected where available. Type of AF was defined as either paroxysmal (self-terminating episodes lasting < 7 days) or persistent (sustained episodes lasting ≥ 7 days).

## Pulsed field ablation procedure

All patients underwent PFA with the Farapulse^™^ system (Boston Scientific, US) under general anaesthesia. Periprocedural anticoagulation strategy was according to physician preference. Femoral venous access was obtained for each patient with a 6Fr and 7Fr sheath. A multipolar catheter was inserted into the coronary sinus for the duration of the procedure in most cases. If patients were in AF at the beginning of the procedure, they were routinely cardioverted prior to performing the transseptal puncture. Transseptal puncture was performed under transesophageal echocardiography and hemodynamic monitoring guidance. Peri-procedural intravenous heparin was administered to achieve a target activated clotting time (ACT) of > 350 s. Atropine was administered to minimise the risk of vagal response. Following transseptal puncture, a J-tipped wire was typically advanced into the left superior pulmonary vein (LSPV) to facilitate sheath cannulation directly with the Faradrive sheath to left upper pulmonary vein, or after the wire and dilator was removed, an Amplatz Extra Stiff guidewire (Cook Medical, USA) was used instead to wire the LSPV and the Faradrive deflectable 13 F sheath (Farapulse^™^) passed over it. Either a 31–35 mm pentaspline catheter was selected based on left atrial size. PFA was typically performed on the pulmonary veins in all cases, and ablation of the posterior wall and other non-pulmonary vein triggers varied according to physician preference. Each pulmonary vein received at least 4 applications in ‘flower’ and ‘basket’ configuration at 2 kV with paired lesions over a Rosen wire (Cook Medical, US) after approximately 30–45° rotation of spline orientation. Pulmonary vein isolation was assessed by loss of electrogram signals at the ostium. Posterior wall ablation was performed with overlapping applications across the posterior wall covering both the superior and inferior aspect with isolation confirmed by loss of PW electrograms after ablation. Additional lesions were applied at operator discretion where there were perceived gaps after intended lesion set. Where available from February 2025 onwards, electroanatomic mapping was concurrently used through an electroanatomic map software integrated into the Farapulse^™^ system (Faraview^™^, Boston Scientific, United States).

Upon completion of PFA, catheters were removed and femoral closure performed with either figure-of-eight suture or a vascular closure device such as Perclose™ ProStyle™ devices (Abbott Vascular, United States) according to physician preference [9]. All patients were monitored in the immediate recovery area. Patients underwent same-day discharge as per standard protocol or were admitted to hospital if necessary or requested by the proceduralist.

Procedural characteristics collected included lesion sets (e.g. pulmonary vein isolation, posterior wall isolation, superior vena cava isolation), total number of lesions, total procedure time and left atrial catheter dwell time.

## Arrhythmic recurrence

Monitoring for arrhythmias at follow-up was performed based on symptom and clinical indication, with standard 12-lead electrocardiogram (ECG) at clinic review. Arrhythmia recurrence was defined as any AF, AFL or AT lasting more than 30 s at follow-up. Detection of arrhythmia occurrence was by 12-lead ECG if persistent, Holter monitoring or intracardiac device check at follow-up, or wearable device.

## Repeat procedure

Repeat procedures were performed under general anesthaesia. The decision to use 3D electroanatomic mapping systems was at operator discretion, as was choice of specific electroanatomic mapping system (CARTO 7, Biosense Webster; Ensite X Mapping, Abbott; Rhythmia, Boston Scientific). Cardioversion was performed prior to mapping if patients presented in AF, while activation mapping was performed for AFL. Confirmation of PVI was reliant on electrograms recorded from a multipolar mapping catheter or the pentaspline PFA catheter. Qualitative assessments concerning isolation, bipolar voltages and scar were also undertaken. If PV reconnection was identified, ablation was performed using either radiofrequency (Thermocool SmartTouch or QDOT Micro, Biosense Webster; Tacticath SE or Tactiflex, Abbott) or PFA (Farapulse, Boston Scientific) at operator preference. Linear ablations were performed at operator discretion for atypical AFL using RF. Bidirectional block and differential pacing was used to evaluate for block when performing cavotricuspid isthmus (CTI) line ablation for typical AFL. Superior vena cava (SVC) isolation was performed using PFA by delivering sequential applications at the site of earliest SVC activation during sinus rhythm with the endpoint being loss of all SVC potentials.

## Meta-analysis

A digital literature search was performed through MEDLINE and EMBASE databases for published articles up to September 2025. Key words using Medical Subject Heading (MeSH), where available, included ‘atrial fibrillation’, ‘pulsed field ablation’, ‘repeat procedure’ and ‘redo ablation’. Reference lists of previous relevant articles were also reviewed for further articles. The inclusion criteria for studies were as follows: (1) evaluation of patients undergoing clinically indicated repeat procedures after index PFA using a specific pentaspline catheter (Farapulse, Boston Scientific) for AF, (2) English language and (3) fully published status. We excluded studies that did not specifically evaluate findings at repeat procedure, studies that evaluated role of PFA as primary ablation modality for repeat procedure after index procedure with thermal ablation and studies that performed routine protocol-driven repeat procedure to evaluate PV durability rather than by clinical indication for arrhythmia recurrence. Data items to be collected were determined prior to the literature search. One investigator (D.P) conducted the literature search independently and extracted data for study design, baseline patient demographics, index procedural characteristics, incidence of repeat ablation, type of arrhythmia recurrence (AF, AFL, AT) and PV and PW reconnection rate where documented. Extracted data were verified by the first author (F.J.H) with any discrepancies resolved by consensus. The study protocol was prospectively registered with the PROSPERO international register (CRD420251172312).

### Endpoints of meta-analysis

End points for the meta-analysis included rate of repeat procedure after index PFA for AF, proportion of patients with any PV reconnections, proportion of PV reconnections compared with total PVs, specific reconnection rates for each PV, and PW reconnection rate.

## Statistical analyses

Categorical variables are presented as frequencies and percentages, while continuous variables are reported as median with interquartile range (IQR) or as mean with standard deviation (SD), depending on whether the data were normally distributed. Data for incidence of repeat procedure, PV reconnection per patient, specific PV reconnection and PW reconnection were analyzed by Freeman-Tukey double arcsine transformation with random-effects modeling. Pooled prevalence was reported as summary estimate (ES) with 95% confidence interval (CI). Chi-squared test was used to compared categorical variables (or.

Fisher’s exact test when count was *<* 20). Logistic regression was used to evaluate the association between variables and need for repeat procedure and reported as odds ratio (OR) with 95% confidence interval (CI). Statistical analyses were performed using Stata MP 19.0 (Stata Corp LP, College Station, TX).

## Results

### Baseline patient characteristics

In a total of 751 index PFA procedures for AF, there were 50 patients who underwent repeat procedure (6.7%; Table 1). The time from index procedure to repeat procedure was 6.8 months (IQR 3.2–10.1). The median age at index procedure was 63 years (IQR 55–67) and 38% (19/50) were female. Twenty-four (48%) had persistent AF as indication for index ablation and a median CHA₂DS₂-VASc of 2 (IQR 1–3).

**Table 1:** Baseline patient and index procedural characteristics

Baseline characteristics	*N* = 50
Age, years	63 (55–67)
Female (%)	19 (38)
BMI, kg/m^2^ (IQR)	29.1 (25.8–35.0)
CHA₂DS₂-VASc, median (IQR)	2 (1–3)
Comorbidities (%)	
Hypertension	25 (50)
Vascular disease	8 (16)
Heart failure	8 (16)
Diabetes mellitus	7 (14)
Stroke/TIA	1 (2)
Type of AF (%)	
Paroxysmal	26 (52)
Persistent	24 (48)
LVEF, % median (IQR)	60 (55–62)
LAVI, ml/m^2^	39 (35–60)
Anti-arrhythmic drug	
Flecainide	17 (34)
Sotalol	16 (32)
Amiodarone	6 (12)
None	11 (22)
Index procedure characteristics	
Pentaspline catheter	
31 mm	39 (78)
35 mm	11 (22)
Integrated electroanatomic mapping	5 (10)
Ablation type	
Pulmonary vein	50 (100)
Posterior wall	23 (46)
Number of PFA applications	
Total applications	56 (46–72)
Pulmonary vein applications	50 (44–54)
Posterior wall applications	21 (18–24)
Procedural time, minutes	62.5 (59–72)
Left atrial catheter dwell time, minutes	43.5 (38-53.5)

*IQR* Interquartile range*, LAVI* Indexed left atrial volume*, LVEF *Left ventricular ejection fraction

### Index ablation characteristics

Most patients underwent index PFA using the 31 mm pentaspline catheter (78%; 39/50) while in 11 patients (22%) the 35 mm catheter was used (Table 1). All patients underwent pulmonary vein isolation successfully, and 23 patients (46%) received posterior wall ablation as well. The total number of applications was 56 (IQR 46–72) with a median of 50 pulmonary vein applications (IQR 44–54) and 21 posterior wall applications (IQR 18–24). The median procedural time was 62.5 min (IQR 59–72 min).

### Recurrent arrhythmia type

The recurrent arrhythmia indication for repeat procedure was AF in 24 patients (48%), AT or atrial flutter in 18 patients (36%) and both forms in 8 patients (16%) (Table 2). There was no difference in the type of recurrent arrhythmia based on what ablation lesion set they received at index procedure (PVI only or PVI and posterior wall; *p* = 0.60). On univariate analysis, the only significant predictor of undergoing repeat procedure was persistent AF as indication for initial index procedure (OR 1.8, 95% CI 1.06–3.18, *p* = 0.03). Notably, age (*p* = 0.99), female sex (*p* = 0.37), hypertension (*p* = 0.92), diabetes mellitus (*p* = 0.33) and LAVI (*p* = 0.37) did not predict likelihood of repeat procedure. Neither the use of integrated 3D electroanatomic mapping (*p* = 0.27) nor device size (31 mm vs. 35 mm pentaspline catheter; *p* = 0.64) predicted repeat procedure.

**Table 2:** Recurrent arrhythmia type and repeat procedure characteristics

Recurrent arrhythmia	*N* = 50 (%)
AF	24 (48)
AT/AFL	18 (36)
Both AF and AFL	8 (16)
Electroanatomic Mapping	
None	3 (6)
Rhythmia	7 (14)
Carto	13 (26)
Ensite X	27 (54)
Ablation modality	
Pulsed field ablation	13/48 (27)
Radiofrequency	35/48 (73)
Additional ablation lesion sets	
Posterior wall	25/48 (52)
Mitral isthmus line	9/48 (19)
Cavotricuspid isthmus	13/48 (27)
Roof line only	6/48 (13)
Superior vena cava	3/48 (6)

*AF *Atrial fibrillation, *AT* Atrial tachycardia, *AFL* Atrial flutter *CTI* Cavotricuspid isthmus *PFA* Pulsed field ablation, *PW *Posterior wall, *RF* Radiofrequency, *SVC *Superior vena cava

### Repeat procedure

At repeat procedure, the most common electroanatomic mapping software used was Ensite X (27/50; 54%) followed by Carto (13/50; 26%). In two patients with AFL, activation pattern on the coronary sinus decapolar catheter suggested likely cavotricuspid isthmus (CTI) dependent flutter and a CTI line was performed with termination of tachycardia without further mapping of the left atrium.

**Fig. 1:**
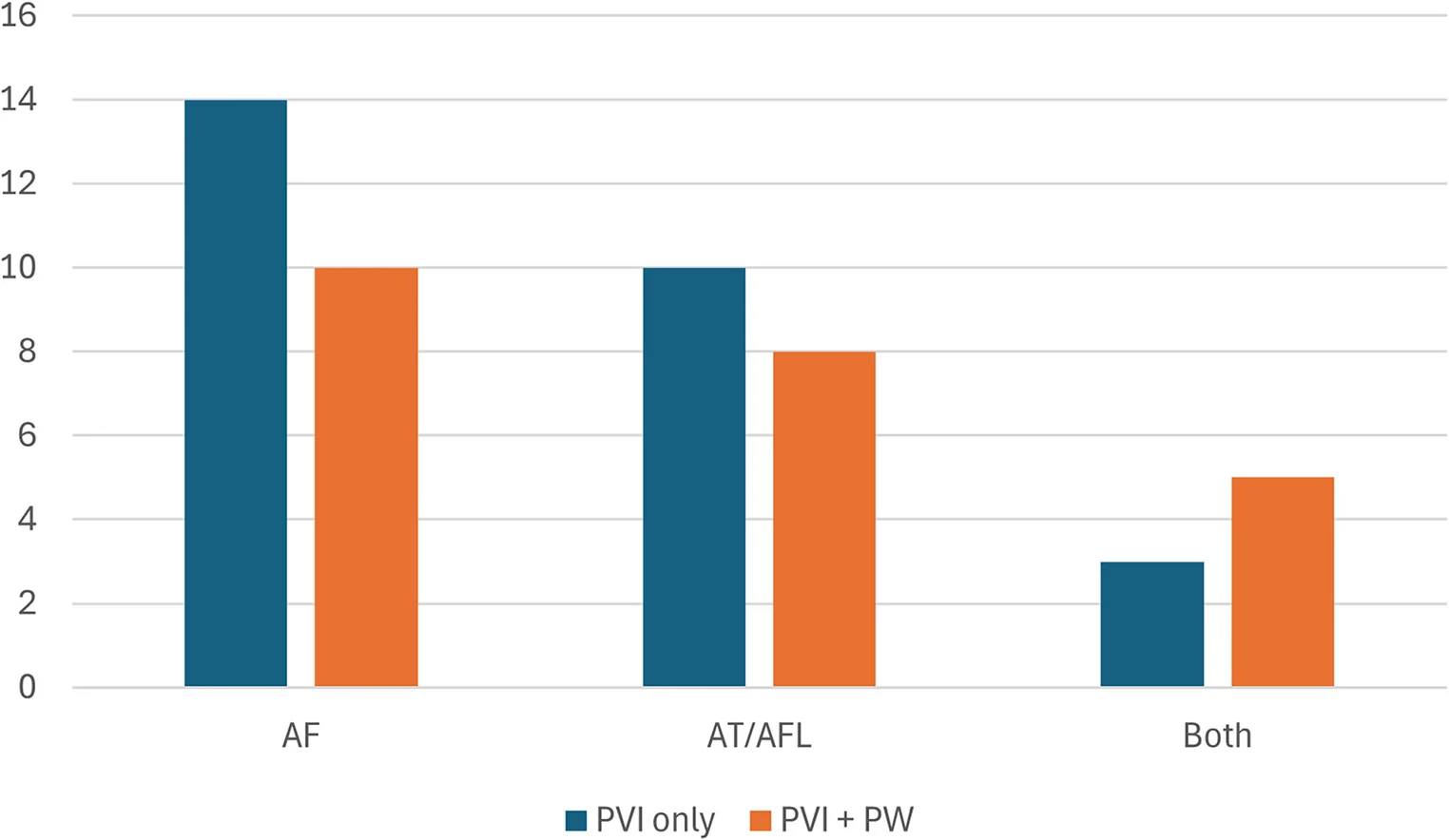
Type of recurrent arrhythmia after index ablation based on initial lesion set

**Fig. 2:**
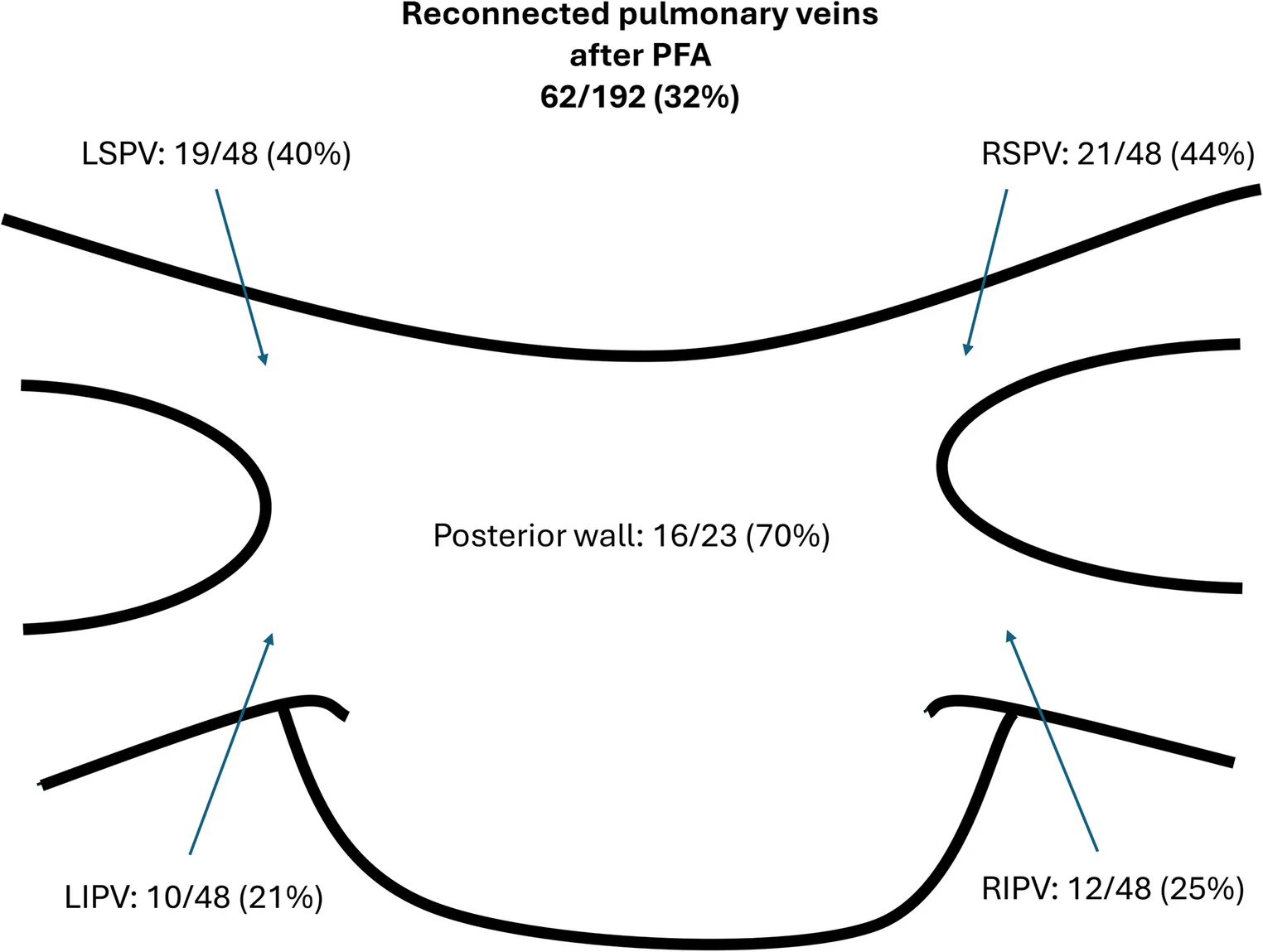
Pulmonary vein and posterior wall reconnection after index pulsed field ablation

Thus in 48 patients, durably isolated PVs was demonstrated in 18 patients (37.5%). In a total of 192 PVs, 130 were durably isolated (68%). The most common reconnection was in the RSPV (44%; 21/48), followed by LSPV (40%; 19/48), RIPV (25%; 12/48) and LIPV (21%; 10/48). The number of reconnected PVs were 0 in 18 patients, 1 in 12 patients (25%), 2 in 10 patients (21%), 3 in 2 patients (4%) and 4 in 6 patients (13%). On Fisher’s exact test, the superior PVs were more likely to be reconnected than the inferior PVs (*p* = 0.005). There was no difference in rate of reconnection overall between right or left sided veins (*p* = 0.64). Visual assessment of PV reconnection suggested more reconnections on the anterior aspect across all the veins (Figure S1 in Data Supplement). There was no association between the number of PFA applications to the PVs on index procedure and likelihood of durably isolated PVs (*p* = 0.27). In patients who underwent PW ablation in their index procedure, the posterior wall was reconnected in 70% (16/23). There was no association between number of PFA application to the PW on index procedure and likelihood of durably isolated PW (*p* = 0.47). In 48 patients who underwent left sided ablation, the ablation modality for repeat ablation procedure was PFA in 13 patients (27%) and RF in 35 patients (73%). Additional lesion sets included PW ablation in 25 patients (52%), mitral isthmus line in 9 patients (19%), CTI lines in 13 patients (27%), roof line in 6 patients (13%) and SVC isolation in 3 patients (6%). Amongst 15 patients who were in atypical AFL at time of repeat procedure, eight patients had peri-mitral AFL, six patients had roof dependent AFL, one patient had non-specific AFL at the inferior aspect of the posterior wall and one patient with dual loop AFL (roof dependent and peri-mitral). There was no significant association between patients who underwent index PV isolation alone (without posterior wall ablation) and roof-dependent AFL (*p* = 0.19). (Figs. 1 and 2).

### Meta-analysis of repeat procedures and reconnections

**Fig. 3:**
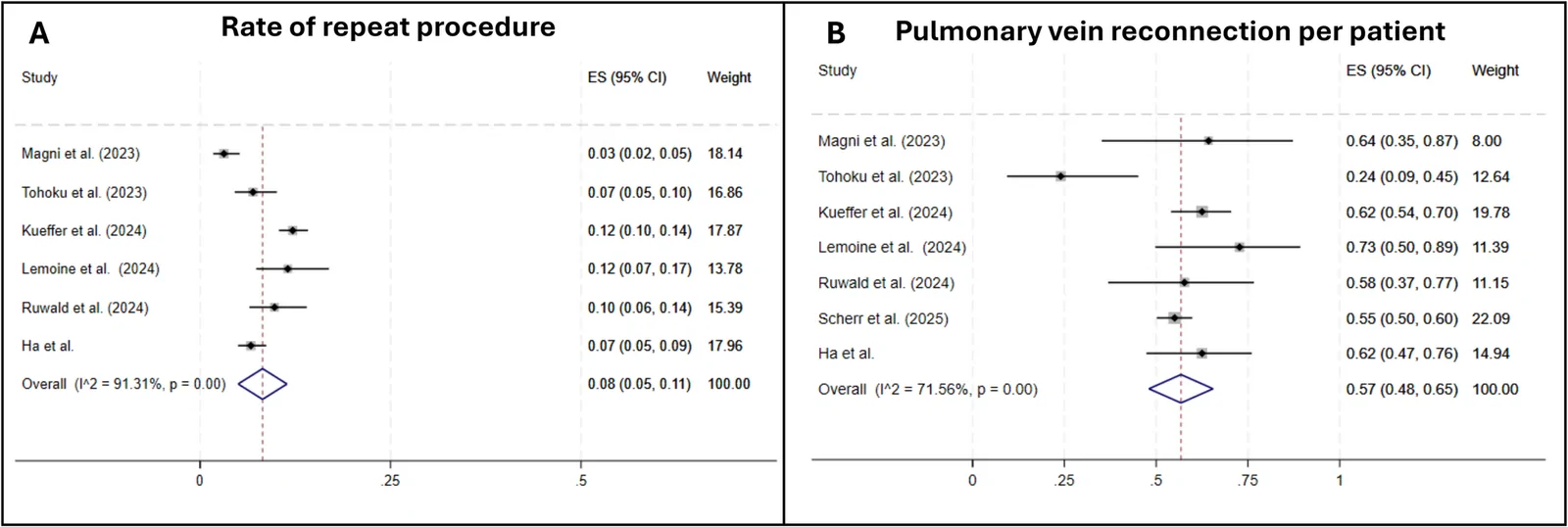
Meta-analysis of rate of repeat procedures (**A**) and pulmonary vein reconnections per patient (**B**)

From a total of 204 studies screened (S2 in Data Supplement), six additional studies with a total of 708 patients (including 50 patients from our present study) underwent a repeat procedure after index PFA for AF (Table 3) [5, 7, 8, 10–12]. Where the total index procedure was reported (6 studies with a total of 3199 patients), the incidence of repeat procedures was (8.8%; 281/3199; Fig. 3). However, there was significant heterogeneity (I^2^ = 91%; *p* < 0.01) in incidence of repeat procedures between studies, ranging from 3% to 12%.

**Table 3:** Baseline patient characteristics, atrial tachyarrhythmia recurrence and pulmonary vein and posterior wall reconnection on repeat procedure in included studies on meta-analysis

Study (year)	Location	No. of patient index PFA	No. of patients repeat procedure	Age, years	Female	Persistent AF before index PFA	Arrhythmia recurrence				Time from index to repeat procedure, months	PV reconnection / total patients (%)	RSPV (%)	RIPV (%)	LSPV (%)	LIPV (%)	PW (%)
							AF	AFL/ AT		Both							
Magni et al. [10]	NetherlandsGermany	447	14	61.9 ± 10.8	50%	50%	50%	14%	36%		NR	9/14 (64)	3/14 (21)	7/14 (50)	4/14 (29)	5/14 (36)	2/3 (67)
Tohoku et al. [5]	Germany	360	25	72 ± 9	64%	24%	32%	68%	NR		6.1 ± 4.0	6/25 (24)	1/25 (4)	2/25 (8)	4/24 (17)	2/24 (8)	NR
Kueffer et al. [7]	Europe	1184	144	68.9 (61.4–73.6)	37.5%	48%	68%	32%	NR		7 (5–10)	90/144 (63)	34/144 (24)	44/144 (31)	43/135 (32)	37/135 (27)	NR
Lemoine et al. [11]	Germany	191	22	71 (65–77)	50%	72%	73%	18%	9%		7.8 (6.3–12.6)	16/22 (73)	5/22 (23)	14/22 (64)	7/22 (32)	7/22 (32)	NR
Ruwald et al. [12]	Denmark	266	26	65 ± 10	58%	65%	65%	35%	NR		9.6 ± 3.9	15/26 (58)	9/26 (35)	11/26 (42)	7/26 (27)	5/26 (19)	1/5 (20)
Scherr et al. [8]	Europe	NR	427	64 ± 11	37%	53%	81%	19%	NR		9.2 ± 5.6	235/427 (55)	141/427 (33)	137/427 (32)	128/427 (30)	120/427 (28)	76/140 (54)
Ha et al.	Australia	751	50	63 (55–67)	38%	48%	48%	36%	16%		6.8 (3.2–10.1)	30/48 (63)	21/48 (44)	12/48 (25)	19/48 (40)	10/48 (21)	16/23 (70)

**Fig. 4:**
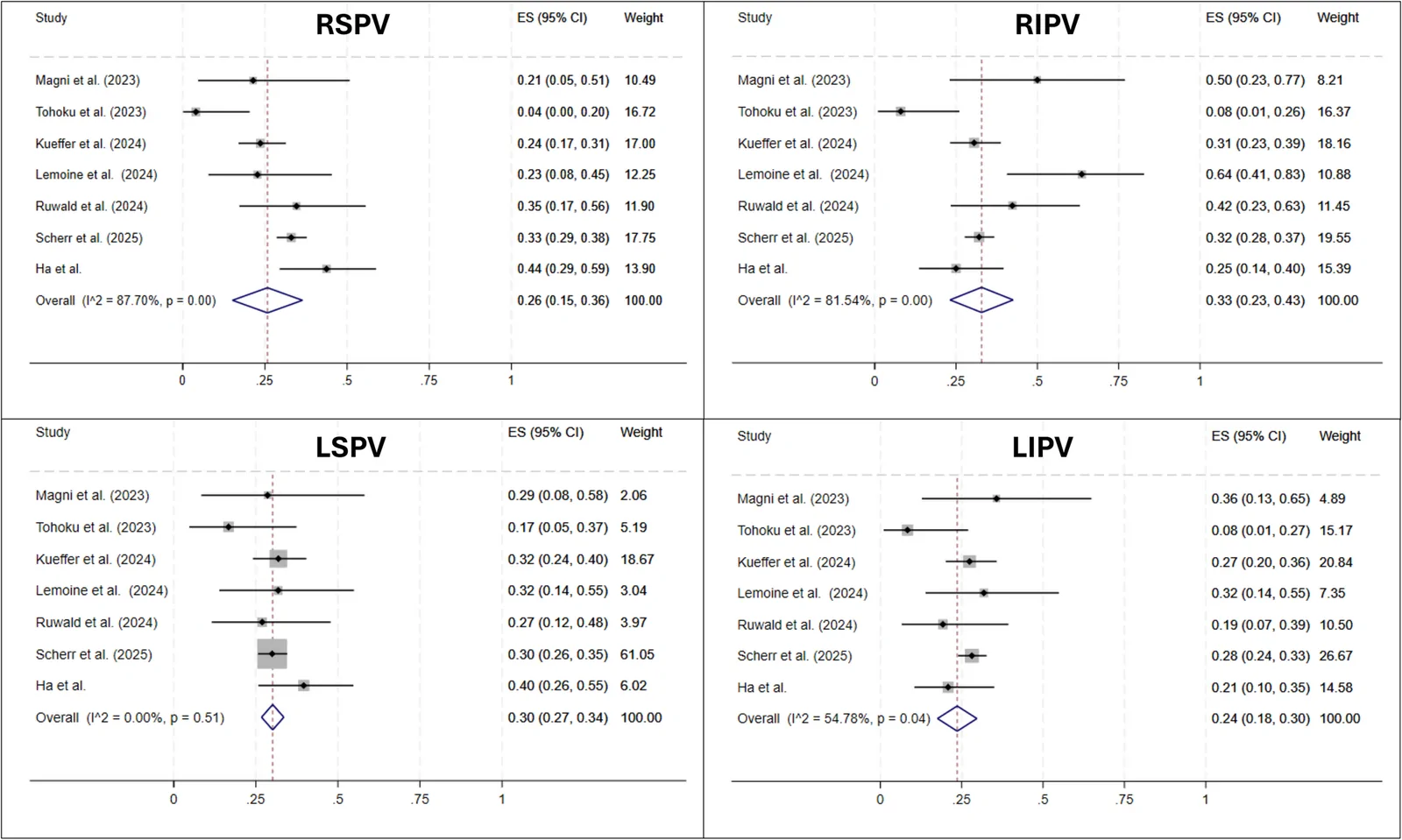
Meta-analysis of reconnections in each pulmonary vein

**Fig. 5:**
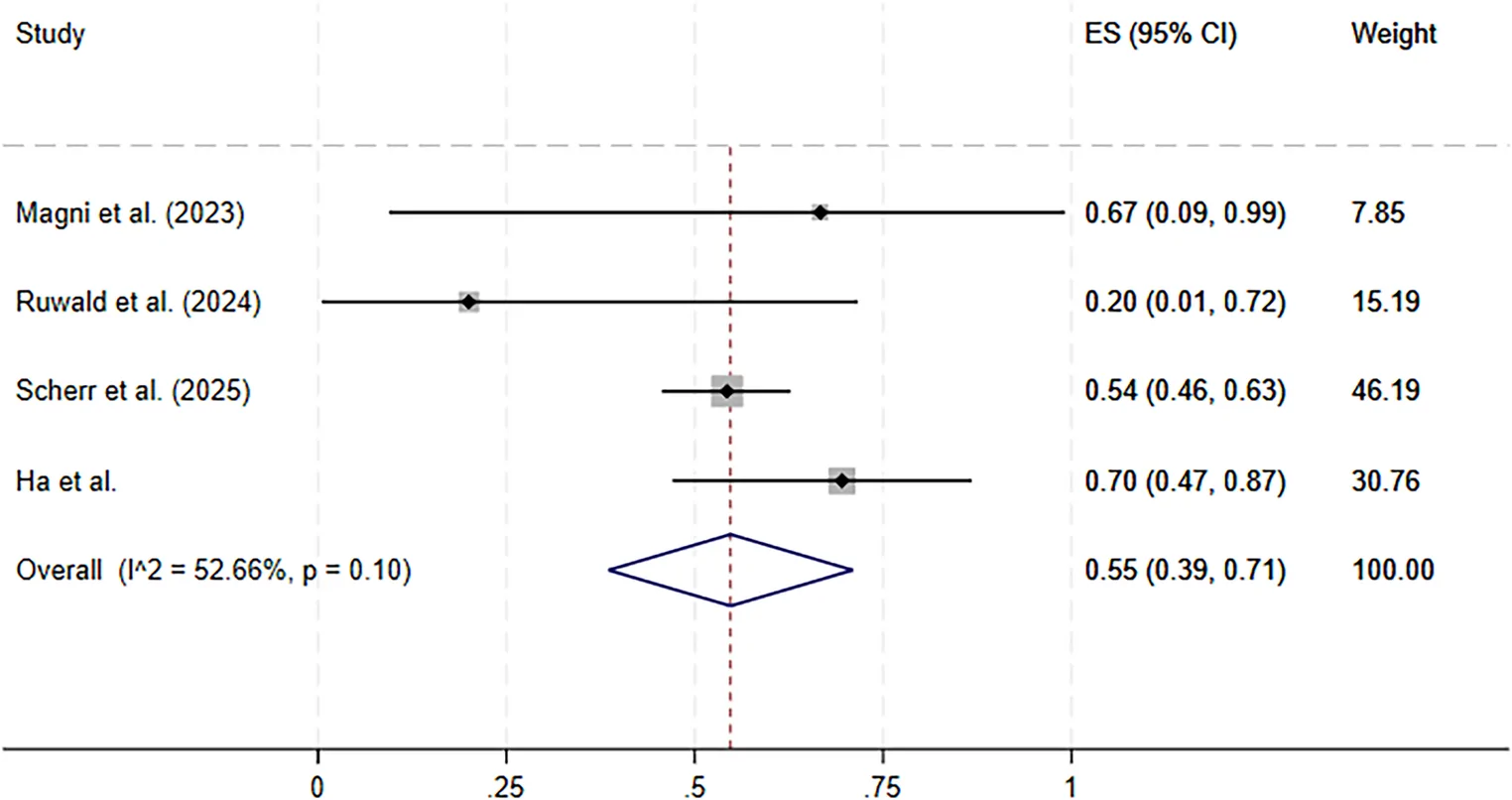
Meta-analysis of reconnection in posterior wall

The overall rate of at least one PV reconnection per patient was 57% (401/706) however with significant heterogeneity between studies (I^2^ = 72%, *p* < 0.01). In evaluation of risk of bias with a leave-one-out analysis, the removal of one study led to a similar rate of 59% but with no heterogeneity (I^2^ = 14%, *p* = 0.32; Table S1 in Data Supplement). Pulmonary vein reconnection per patient ranged from 24% to 73% (Fig. 3). In relation to each individual PV, on meta-analysis (Fig. 5), the RSPV reconnection was 26% (95% CI 0.15–0.36; I^2^ = 88%), RIPV reconnection was 33% (95% CI 0.23–0.43; I^2^ = 81%), LSPV reconnection was 30% (95% CI 0.27–0.34; I^2^ = 0%) and LIPV reconnection was 24% (95% CI 0.18–0.30; I^2^ = 55%). On meta-analysis of four studies that reported PW reconnection (Fig. 4), the reconnection rate 55% (95% CI 0.39–0.71) with non-significant heterogeneity (I^2^ = 53%; *p* = 0.10).

## Discussion

We performed an observational study of consecutive patients who underwent repeat procedure after index PFA for AF at our tertiary centre. Additionally, we performed a meta-analysis of published observational studies that similarly evaluated repeat procedures after PFA with the same pentaspline catheter. We found that.

1. Amongst 751 index PFA procedures, the repeat procedure rate at our centre was 6.7% which appears largely comparable with other studies ranging from 3 to 12%.
2. The PV reconnection rate on a patient-level analysis in our centre was 62% which is in keeping with most observational studies with an incidence of 57% (95% CI 48%-65%) on meta-analysis despite heterogeneity.
3. Superior PVs were significantly more likely to be reconnected (40–44%) on either the left or right side compared with inferior PVs (21–25%; *p* = 0.005 for difference).
4. Reconnection of the PW after PFA was high (70%) and is largely consistent with previous studies albeit with few cumulative patients (*n* = 171) but no significant heterogeneity.

The advent and rapid uptake of PFA globally has changed the way we treat AF and the threshold to offer ablation. While early data appears promising in avoiding injury to non-cardiac structures such as the oesophagus, [13–15] longer term real-world outcomes are still lacking alongside lesion durability [16]. Offering a repeat procedure to patients with symptomatic arrhythmia recurrence must be thoughtfully considered; not only in view of the invasive nature and potential risk of complications, but the documented diminishing returns of multiple repeat procedures. Indeed, while freedom from AF might improve following the second ablation procedure in the era of thermal ablation, there is minimal difference when pursuing a third procedure [17]. Yet the need for repeat procedures after index PFA appears numerically and anecdotally less compared with thermal ablation at short to medium term follow-up. Repeat procedures after thermal ablation has been up to 26% at 5-year follow-up,^17^ while our meta-analysis shows a real-world repeat procedure rate of 8.8% after index PFA. In a retrospective analysis of the FIRE AND ICE trial of patients who underwent re-ablation for symptomatic recurrences, the repeat procedure rate was 11.7% after about 6 months follow-up with between median one to two reconnected veins. The reconnection rates were significantly higher with initial radiofrequency compared with cryoballoon ablation [18]. While PFA modifies AF substrate leading to less AF burden and symptomatic recurrences, longer term follow-up for this technology is still warranted.

PV reconnection rates after index PFA have been variable in observational studies thus far. While an early small study reported PV reconnections in only 24% of patients at repeat procedure at 6.1 months, this is significantly lower than other published studies leading to statistical heterogeneity on meta-analysis. Indeed, when a risk of bias analysis by sequential removal of each individual study was performed, there was no longer significant heterogeneity in the pool estimate. These disparate findings may be reflected by a small sample size, operator experience, few patients (24%) having a more persistent form of AF prior to index ablation and the higher proportion of macro-reentrant AT/AFL (68%) as opposed to AF as the indication for repeat procedure. Nevertheless, other real-world experience including those from larger registries have not replicated such PV durability and highlights the ongoing need to improve procedural strategies for PFA. Data from MANIFEST-REDO suggested that previous use of imaging or mapping modality was univariately associated with durable PVI [8]. We did not find an association between integrated mapping from the pentaspline catheter (Faraview, Boston Scientific) at index PFA and PV durability although only five patients who underwent repeat procedure had integrated mapping available at index procedure and this finding is likely hampered by type II error. Preclinical data from a swine-beating heart model has also shown the importance of contact force for increasing lesion depth at constant PFA current intensity [19]. Such information during index PFA is likely to become standard as PFA technology evolves. This is particularly evidenced by the high rate of posterior wall reconnection even with PFA in our experience (70%) and meta-analysis (55%). Furthermore, certain patient factors may need to be accounted for, whereby PVI durability is likely poorer in those with significant left atrial scar or dilatation [20]. 

Our study found that the superior PVs were more likely to be reconnected than the inferior PVs which was statistically significant. This may be due to inadequate contact from the catheter splines when directed superiorly under fluoroscopic guidance. This could be overcome with the aforementioned use of impedance-based contact information as well as intracardiac echocardiography although the routine use of such imaging is limited at our centre due to financial constraints. Additionally, on visual assessment of segments of the PV and carina, the anterior aspects were more frequently reconnected than the posterior aspects of veins. These findings are not dissimilar to previous published data in repeat procedure outcomes after PFA where reconnections tended to be more anterior, especially in the RIPV [11]. On the left sided PVs, anterior reconnections may be in part due to the proceduralist caution about delivering PFA applications in proximity to the left atrial appendage with risk of isolation and potential thrombus formation [21]. On the right sided PVs, anterior reconnections could be explained by initial transseptal puncture whereby an anteriorly directed transseptal crossing can often lead to difficulties manipulating the catheter to the anterior aspect of the right sided PVs, especially with the larger 35 mm catheter. Such procedural considerations should be taken into account given the above findings for more likely areas for PV reconnections.

We acknowledge several limitations in this study. First, this is a single centre retrospective analysis of real-world clinical outcomes. As such, operator experience and preference for ablation strategy and need for repeat procedure could differ with other centres. Second, given repeat procedures were driven by clinical arrhythmia recurrence rather than routine protocol driven evaluation (as might be in prospective study design), it is likely that the overall PV and PW durability is underestimated. Thus the overall PV and PW durability is expected to be higher in the overall cohort including those without arrhythmic recurrences.

## Conclusions

The repeat procedure rate after index PFA for AF at our centre is relatively low (6.7%) and consistent with published observational studies in this meta-analysis (8.8%). However, PVI and PW durability were 43% and 55%, respectively, on meta-analysis. Recognising patient factors and ongoing procedural and technological development to PFA is needed to improve lesion durability.

## Supplementary Information

Below is the link to the electronic supplementary material.

Supplementary Material 1 (DOCX 697 KB)

## Data Availability

Data are available on request.
