# Correction: Repeat procedures after pulsed field ablation for atrial fibrillation: tertiary centre experience and meta-analysis of observational studies

**DOI:** 10.1007/s10840-026-02287-w

**Published:** 2026-03-05

**Authors:** Francis J. Ha, Sarah Paitry, Duron Prinsloo, Hui-Chen Han, Adam J Brown, Nitesh Nerlekar, Emily Kotschet

**Affiliations:** Victorian Heart Institute and Victorian Heart Hospital, Monash University, Monash Health, 631 Blackburn Road, Clayton, VIC 3168 Australia

**Correction to**:** Journal of Interventional Cardiac Electrophysiology**

**https://doi.org/10.1007/s10840-026-02258-1**

In this article, the captions for Figs. 4 and 5 were inadvertently interchanged. The correct captions are as follows:

**Fig. 4** Meta-analysis of reconnections in each pulmonary vein

**Fig. 5** Meta-analysis of reconnection in posterior wall

The original article has been corrected.

