# Supplementary material for: Repeat procedures after pulsed field ablation for atrial fibrillation: tertiary centre experience and meta-analysis of observational studies
Source: J Interv Card Electrophysiol. 2026 Feb 19;69(7):1837–46. doi: 10.1007/s10840-026-02258-1 (PMC13687113; doi:10.1007/s10840-026-02258-1)
Supplement: Supplementary file 1 [file 10840_2026_2258_MOESM1_ESM.docx]

**Repeat Procedures after Pulsed Field Ablation for Atrial Fibrillation:
Tertiary Centre Experience and Meta-Analysis of Observational studies.**

Supplementary materials

**Figure S1. PV reconnection by anatomical location**

**Figure S2. PRISMA flow diagram**

**Table S1. Leave-out-one analysis. PV reconnections per patient**

| Study excluded | ES (95% CI) | Heterogeneity, I^2^ | P-value for heterogeneity |
| --- | --- | --- | --- |
| Magni et al. (2023) | 0.56 (0.47-0.65) | 76% | P<0.01 |
| Tohoku et al. (2023) | 0.59 (0.55-0.64) | 14% | P=0.32 |
| Kueffer et al. (2024) | 0.55 (0.44-0.67) | 73% | P<0.01 |
| Lemoine et al. (2024) | 0.55 (0.46-0.64) | 72% | P<0.01 |
| Ruwald et al. (2024) | 0.57 (0.47-0.66) | 76% | P<0.01 |
| Scherr et al. (2025) | 0.57 (0.45-0.70) | 75% | P<0.01 |
| Ha et al. | 0.56 (0.46-0.66) | 75% | P<0.01 |
